# Haplotype-resolved T2T genome assembly of the pear cultivar ‘Danxiahong’

**DOI:** 10.1038/s41597-025-05380-3

**Published:** 2025-06-18

**Authors:** Xiangzhan Zhang, Jianxin Bian, Yanan Wang, Lei Wang, Long Wang, Suke Wang, Yanli Su, Hang He, Huabai Xue

**Affiliations:** 1 National Key Laboratory for Germplasm Innovation & Utilization of Horticultural Crops, Zhengzhou Fruit Research Institute, Chinese Academy of Agriculture Sciences, Zhengzhou, 450009 Henan China; 2 Zhongyuan Research Center, Chinese Academy of Agriculture Sciences, Xinxiang, 453500 Henan China; 3 Peking University Institute of Advanced Agricultural Sciences, Shandong Laboratory of Advanced Agricultural Sciences in Weifang, Weifang, 261325 Shandong China; 4 College of Horticulture, Xinjiang Agricultural University, Urumqi, 830052 China

**Keywords:** Haplotypes, Comparative genomics

## Abstract

Pear (*Pyrus* L) is one of the most significant fruit crops globally, recognized for its substantial economic value and potential health benefits. ‘Danxiahong’ is an elite pear cultivar in the north of China, characterized by its flushed fruit skin and excellent inner quality. In this study, we utilized PacBio HiFi long reads, Hi-C reads and second-generation sequencing data to assemble the genome of ‘Danxiahong’. Two telomere-to-telomere gap-free and haplotype-resolved pear genomes were successfully assembled, with the sizes of 495.37 Mb and 501.60 Mb, and contig N50 of 28.97 Mb and 29.32 Mb. Approximately 62.50% and 62.76% repeat sequences were mapped to the 17 chromosomes for each haplotype. Gene annotations analysis identified a total of 39,936 genes in Hap1 and 39,707 genes in Hap2, respectively. The haplotype-resolved genome of ‘Danxiahong’ significantly contributes to the investigation of genes and molecular mechanisms related to fruit quality, while also facilitating the Multi-Omics analysis, such as comparative genomics, transcriptomics, proteomics, and allelic expression research.

## Background & Summary

Pear (*Pyrus* L.) is a member of the *Rosaceae* family and *Pyrus* genus, originating from southwest China. It is an important temperate fruit crop in China and is extensively cultivated worldwide, with an ancient cultivation history of more than 3000 years^1,2^. As a globally significant fruit crop with considerable economic and nutritional importance, annual pear production has reached approximately 26.32 million tons in 2022, covering a harvested area of 14.18 million hectares worldwide. In China alone, production accounted for 19.37 million tons (harvested area of 10.05 million hectares), (FAOSTAT https://www.fao.org/faostat/en/#data/QCL), accounting for more than 70% of the global total and ranking first globally.

More than 20 *Pyrus* species have been recognized by most taxonomists, however, only a limited number of *Pyrus* species are commonly cultivated on a global scale. It is generally acknowledged that two primary *Pyrus* groups are predominantly cultivated worldwide, including European pears and Asian pears^3,4^. These two groups exhibit distinct geographical distributions, leading to the prevailing consensus that they have followed independent evolutionary routes^1^. This notion is further supported by phylogenetic analysis utilizing various molecular markers and DNA sequences, which confirmed the separate evolutionary paths taken by Asian and European pears^5^.

Numerous fruit tree species, including pears, are characterized by prolonged juvenility period, self-incompatibility, and high heterozygosity. These traits present significant challenges in gene mining of complex traits^6^. However, with the rapid advancement of technologies, such as high-throughput sequencing, integrated omics approaches, advanced molecular techniques, and specialized bioinformatics tools, substantial progress has been made in genetic research related to various pear traits, including fruit color^7^, stone cell^8^, self-compatibility^9^, bud dormancy^10^, and other agronomic traits.

Given the significant economic importance of pear fruits, the genome of several *Pyrus* species have been sequenced and published. ‘Suli’ (*Pyrus pyrifolia*)^11^ as the first sequenced pear genome using whole-genome shotgun strategy, and followed by the European pear cultivar ‘Bartlett’ (*Pyrus communis*)^12^, wild Asian pears ‘Shanxi Duli’ (*Pyrus betuleafolia*)^13^, dwarfing pear rootstock ‘Zhongai 1’ [(*Pyrus ussuriensis* × *communis*) × spp.]^14^, ‘Cuiguan’ (*Pyrus pyrifolia*)^10^, ‘Yunhong No.1’ (*Pyrus pyrifolia*)^15^, and ‘Yuluxiang’^16^. Despite the publication of several *Pyrus* species genomes, there remains a need for more comprehensive genomic resources due to their diverse genetic backgrounds and the lack of haplotype-resolved accurate genome assemblies. Consequently, these factors limit effective gene mining efforts in this economically important genus.

In this study, we utilized the elite red flushed pear cultivar ‘Danxiahong’ for genome assembly. We integrated advanced technologies including PacBio-HiFi, ONT ultra-long, and Hi-C to achieve telomere-to-telomere (T2T) and gap-free genomes. The haplotype-phased genomes consist of 17 contiguous sequences for each haplotype, with genome size of 495.37 Mb and 501.60 Mb, respectively, N50 values of 28.97 Mb and 29.32 Mb. Various tissues, including floral receptacle, flesh, young shoots, tender leaves, and flower petal were sampled for gene identification. Different methods including ab, homologs-and transcript-based identification applying the PASA pipeline. The T2T genome not only provide the comprehensive reference genomic information, but also establishes a robust foundation for the investigating functional genomics in pears.

## Materials and Methods

### Sample collection and genome sequencing

Different tissues of the pear cultivar ‘Danxiahong’ (*Pyrus* L), including tender leaves, young shoots, floral receptacle, flower petal and flesh were sampled from the ten-year old ‘Danxiahong’ trees in the orchard of Zhengzhou Fruit Research Institute (34°72′N 113°71′E), Chinese Academy of Agricultural Sciences, Zhengzhou, China in March and August 2023, respectively. The samples were immediately treated with liquid nitrogen. The tender leaves were utilized for PacBio HiFi, ONT and Hi-C library construction and sequencing, the different tissues were employed for transcriptome analysis.

### DNA isolation, Library construction and sequencing

The high-quality genomic DNA was isolated using the tender leaves of ‘Danxiahong’ following the modified protocol based on the cetyltrimethyl ammonium bromide (CTAB) method^17^. RNA isolation was performed using TRIzol reagent (Invitrogen, Carlsbad, CA, USA) in accordance with the manufacturer’s instructions. The quality of both DNA and RNA was assessed by NanoDrop2000 spectrophotometer (Thermo Fisher Scientific, USA).

For second-generation data, the DNBSEQ-T7 sequencing platform (BGI, Shenzhen, China) was utilized. The raw short reads were filtered using the SOAPnuke software (v2.1.0)^18^ to filter out low low-quality paired reads and obtain clean data based on the following parameters: -lowQual = 20, -nRate = 0.005, -qualRate = 0.5. Ultimately, a total of 450,756,266 reads corresponding to 67.61 Gb clean data were acquired (Table 1).

**Table 1:** Statistic of sequencing data for pear cultivar ‘Danxiahong’ genome assembly.

Library type	Reads number	Total length (Gb)	N50 length (bp)	GC Content (%)	Q30 (%)	Coverage^*^
BGI Data	450,756,266	67.61	2×150	37.2	94.8	137×
PacBio HiFi	3,530,058	60.21	17,260	37.6	99.9	122×
Hi-C	212,714,625	63.43	2×150	38.37	93.42	129×
ONT	348,297	34.01	100,001	—	—	69×

*The coverage was calculated based on the primary genome assembly of 491.85 Mb.

For PacBio HiFi (high-fidelity) sequencing, a standard HiFi library was prepared in accordance with the SMRTbell Express Template Prep Kit 2.0 manual (Pacific Biosciences, CA, USA). A total of 20 μg DNA per sample was utilized for the preparation of DNA libraries. Sequencing was conducted on a Pacbio Sequel II platform. A total of 60.21 Gb HiFi data and 3,530,058 reads were yield, with reads N50 of 17,260 bp (Table 1).

For ONT ultra-long sequencing, the library was prepared using the SQKULK001 kit, following the standard protocol. The purified library was sequenced using a PromethION sequencer (Oxford Nanopore Technologies, Oxford, UK). A total of 36.07 Gb ONT ultra-long reads were yield, with reads N50 of 100,001 bp.

For Hi-C assays, paraformaldehyde was utilized for cell crosslinking to maintain DNA conformation and structure. The cells were lysed using the digestion of restriction enzyme MboI to generate sticky ends. Subsequently, biotin-14-dCTP was introduced and incorporated at the end of oligonucleotides during DNA repair processes biotin-14-dCTP was introduced and incorporated at the end of oligonucleotide during DNA repairing. The resulting DNA fragments were ligated with DNA ligase. The proteins were digested to release the cross-linked state with DNA, after which the labeled DNA was purified and randomly sheared into fragments of 300~500 bp. The biotin-containing DNA fragments were captured and PCR-enriched to construct a Hi-C library. The library was sequenced on the DNBSEQ-T7 platform following the PE150 strategy. A total of 63.43 Gb Hi-C clean data was generated, corresponding to 129 × coverage of the estimated genome size (Table 1).

Isoform sequencing (iso-seq) was employed to generate high-quality transcriptome data from pear cultivar ‘Danxiahong’. Total RNA was isolated from tender leaves, young shoots, floral receptacle, flower petal and flesh. Full-length cDNA libraries were prepared using the SMARTer PCR cDNA Synthesis Kit (Clontech Laboratories, Inc., USA) and sequenced on a PacBio Sequel II platform. A total of 50.36 Gb subreads base was yield with subreads number of 27,021,170.

### Genome survey

**Fig. 1:**
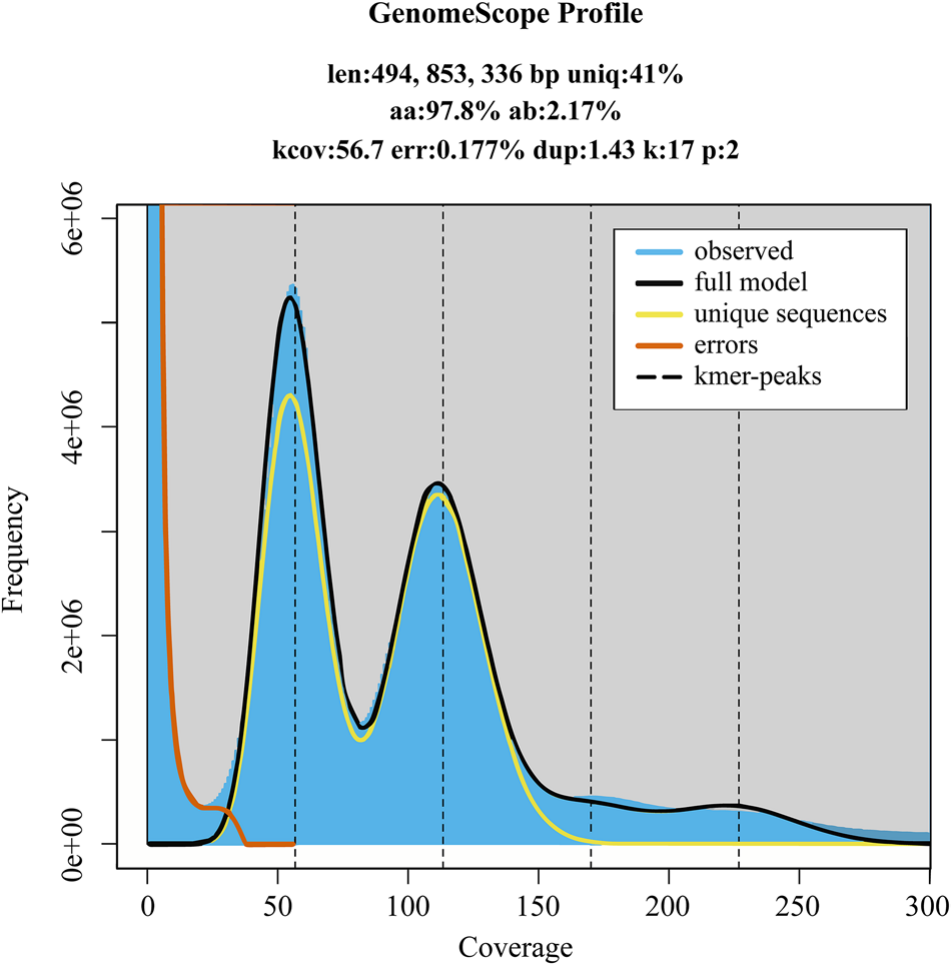
Genome survey of pear cultivar ‘Danxiahong’ based on K-mer analysis using GenomeScope2. Genome size was estimated to be 494.85 Mb, with heterozygosity rate of 2.17%, and a duplication rate of 1.43%.

Genome survey was performed based on the data derived from the BGI MGISEQ platform. Raw data with adapters and low-quality reads were trimmed with SOAPnuke (v2.1.0)^18^ to remove adapters and low-quality reads following the parameters: -lowQual = 20, -nRate = 0.005, -qualRate = 0.5. Then the K-mer analysis was carried out utilizing Jellyfish (v2.2.6)^19^. The genome size, heterozygosity rate and repetition rate were estimated using GenomeScope (v2.1.0)^20^. The result indicated the genome of pear ‘Danxiahong’ was 491.85 Mb, with the heterozygosity rate of 2.17% (Fig. 1).

### Haplotype-resolved genome assembly

Genome assembly was performed using HiFi, ONT reads, and Hi-C data with Hifiasm software (v0.19.9-r616)^21^ using specific command line parameters: hifiasm–ul < ONT data > –h1 < hic_1.fq > –h2 < hic_2.fq >  < HiFi data > . Given the high heterozygosity rate of pear cultivar ‘Danxiahong’ genome, purge_dups (v1.2.3)^22^ was applied to remove duplicated sequences in both haplotypes. A total of 17 chromosomes were assembled for each haplotype. The contig N50 values were 28.97 Mb for Hap1 and 29.32 Mb for Hap2, respectively.

### Hi-C assisted assembly and polishing

**Fig. 2:**
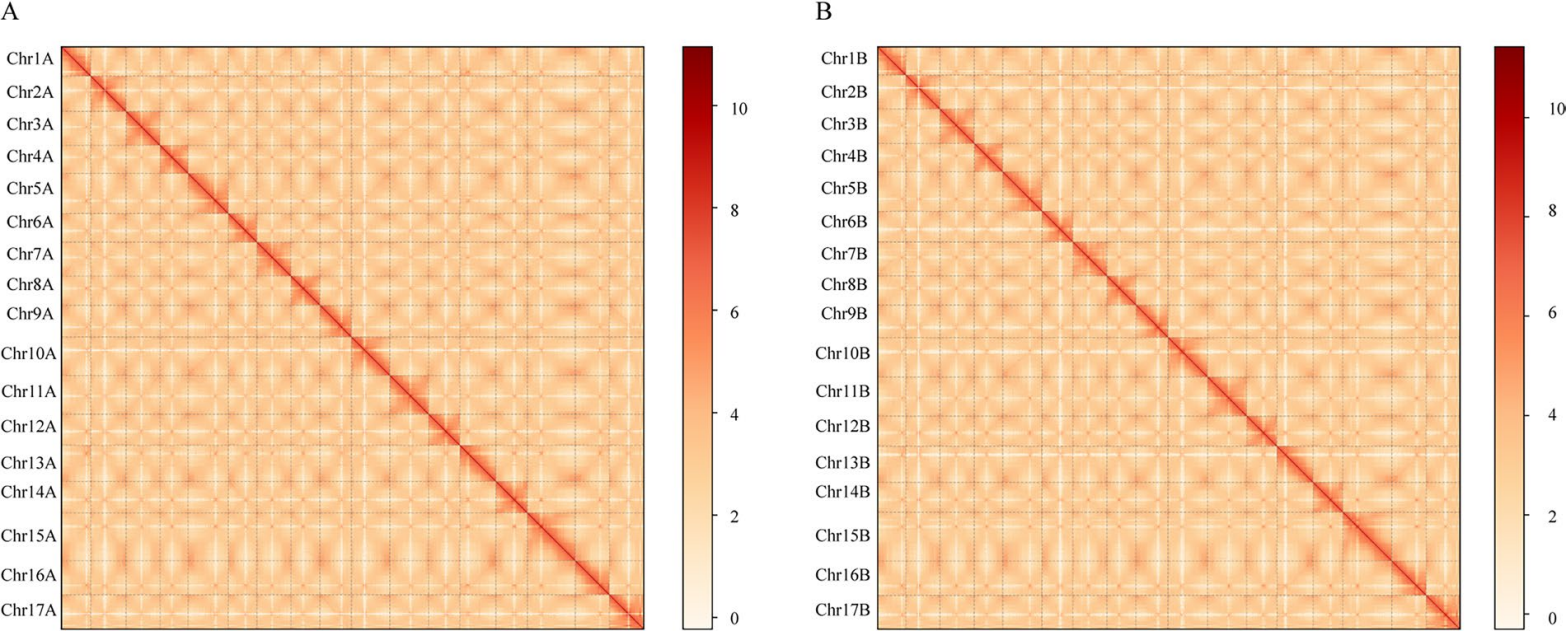
Hi-C interaction heat map for Hap1 (**A**) and Hap2 (**B**) of pear cultivar ‘Danxiahong’ genome assembly.

The high-quality paired-end reads were subjected to Trimmomatic (v0.39)^23^ to remove low-quality bases and adapter sequences. The filtered reads were aligned to draft genome using Juicer (v1.6)^24^ (https://github.com/aidenlab/juicer) to calculate the contact frequency. Subsequently, 3D-DNA (v180922)^25^ was employed with two iterative rounds for misjoin correction (-r1), applying default parameters for clustering and generating an interaction matrix. The oriented scaffolds and contigs were utilized to generate the interaction matrices with a Juicer, allowing for inspecttion and manually corrections with Juicebox (v1.11.08) assembly tools. The error-joins were corrected, the duplicated contigs were removed, resulting in and the generation a primary chromosome-level genome assembly of pear cultivar ‘Danxiahong’ (Fig. 2).

**Fig. 3:**
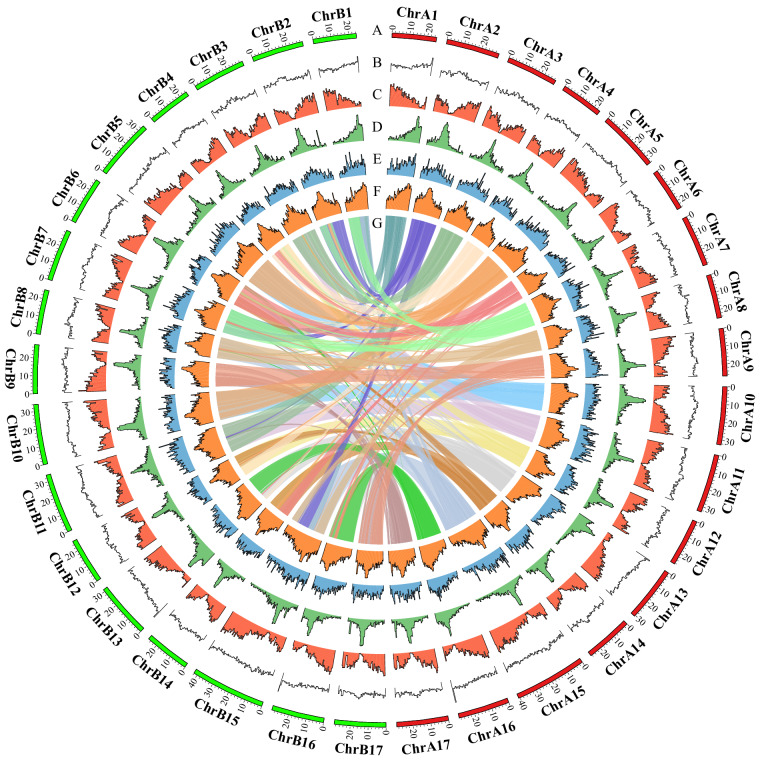
Genomic features of haplotype-resolved genome assembly for the pear cultivar ‘Danxiahong’. Tracks from the outermost to the innermost represents (**A**) assembled 17 pseudochromosomes (Hap1 in red and Hap2 in green), (**B**) GC content, (**C**) gene density, (**D**) *Gypsy*-type retrotransposon, (**E**) *Copia*-type retrotransposon, (**F**) transposon, (**G**) and intragenomic collinearity blocks (>100 kb). The densities of genes, *Gypsy* elements, *Copia* elements, and transposon elements were calculated in 500 kb nonoverlap windows.

The final genome assembly comprised two distinct haplotypes, designated haplotype 1 (Hap1, 495.35 Mb) and haplotype 2 (Hap2, 501.58 Mb), with each containing a total of 17 pseudochromosomes (Fig. 3), respectively. Notably, there were no gaps for each haplotype, indicating the high quality of the T2T no gap haplotype resolved genome assembly (Table 2).

**Table 2:** Statistics of pear cultivar ‘Danxiahong’ genome assembly data.

Items	Hap1	Hap2
Total sequence length (bp)	495,365,661	501,604,687
Number of chromosomes	17	17
Number of contigs	17	17
Contig N50 (bp)	28,972,434	29,324,728
Number of gaps	0	0
GC content (%)	37.34	37.38
Telomeres annotated	34/34	34/34
Number of genes	39,936	39,707
Total TE (bp)	305,968,967	311,090,894
LAI	21.94	21.28
BUSCO (%)	98.82	98.57
QV	40.6567	40.6569

### Detection of telomere and centromere

**Fig. 4:**
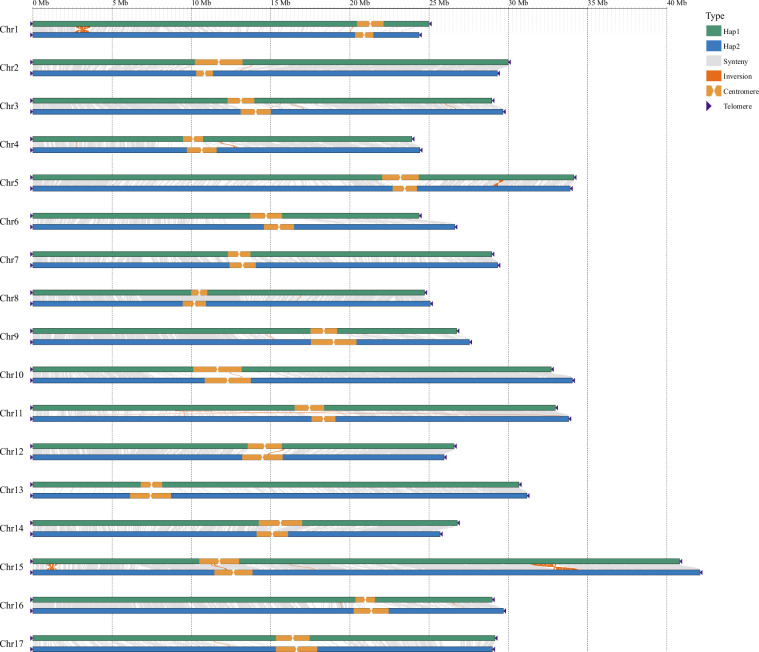
The collinearity analysis and structural variants between Hap1 and Hap2. The collinear regions between the two haplotypes are indicated by gray lines, the inversion regions are denoted with red lines, the centromere regions are marked with orange blocks, and telomeres are indicated by purple triangles.

For the identification of telomeres, the plant telomeric repeat sequences (CCCTAAA/CCCTAAA) were used to identify the telomere regions within 20 Kb for both end of each chromosome using the VGP telomere identification pipeline (https://github.com/VGP/vgp-assembly). In addition, telomeric reads or contigs (including HiFi and ONT reads) were manually determined, and the telomeric sequences were restored for the chromosomes which exhibiting telomere deletions. A total of 34 telomeres (17 chromosomes) for each haplotype were identified (Table 2, Fig. 4).

For centromeres identification, Tandem Repeats Finder^26^ and pyTanFinder (https://github.com/Kirovez/pyTanFinder)^27^ were utilized to identify regions enriched with centromeric tandem repeats clusters. Then the Hi-C interaction heatmap and candidate centromeric tandem repeats were integrated to identify the centromeric regions, and 17 centromeres were identified for each haplotype (Table 2, Fig. 4).

### Genome collinearity analysis

**Fig. 5:**
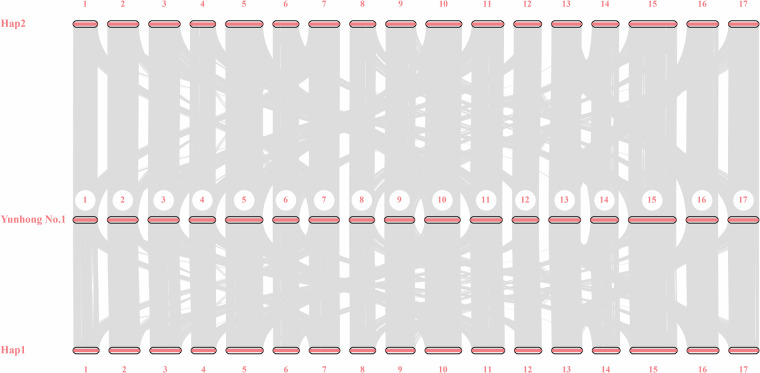
Genome collinearity between the two haplotypes of pear cultivar ‘Danxiahong’ and ‘Yunhong No.1’. Syntenic blocks are highlighted with grey lines connecting different chromosomes, Numbers around the rectangles indicate the chromosomes of each genome.

The collinearity analysis between two haplotypes was performed using mummer (v4.0.0rc1)^28^ applying genome data with delta-filter parameters of -i 95 -o 95 -1. The collinearity between two haplotypes was visualized using GenomeSyn (v1)^29^ with default parameters (Fig. 4). Further, to ensure comprehensive validation, additional collinearity analyses was performed using MCScan (Python version) for the genome of pear cultivar Yunhong No. 1 and Hap1 and Hap2 assemblies. The result demonstrated remarkable collinearity patterns among the different haplotypes and Yunhong No. 1 (Fig. 5).

### Gene prediction and annotation

Three strategies were employed for the prediction of the coding gene structures, including homology-based prediction, transcriptome-assisted prediction, and *ab initio* prediction. For homology-based prediction, protein sequences from closely related species, including *P. communis*(Bartlett), *P. pyrifolia*(Cuiguan), *P. ussuriensis* × *communis* (Zhongai1), *P. pyrifolia* (Nijisseik) and *P. betufolia* (Shanxiduli), were utilized. Tblastn (v2.11.0+) was employed to perform alignment analysis with homologous proteins, and Exonerate (v2.4.0)^30^ was used to identify gene structure. For transcriptome-assisted prediction, the HiSat2 (v2.2.1)^31^ was applied to map the transcriptome data to the genome sequence, then the resulting alignments were assembled into transcripts using genome-guided transcriptome assembler Stringtie (v2.1.7)^32^. Additionally, RNA-seq reads was assembled for accurate *de novo* reconstruction employing the software Trinity (v2.8.5)^33^. An integrated transcriptome database was established, encompassing all transcripts derived from the RNA-seq and Iso-seq data based on analyses conducted with the PASA pipeline (v2.4.1)^34^ analysis. For *ab initio* prediction, different prediction programs, including Augustus (v3.4.0)^35^ and GlimmerHMM (v3.0.4)^36^ were employed to perform *ab initio* gene predictions. Based on the predicted genes obtained from above methods, MAKER (v3.01.03)^37^ was applied to integrate the gene sets into a more comprehensive and non-redundant gene set. Finally, PASA (v2.4.1) was used to update gene structures in accordance with the transcriptome data.

In total, 39,936 protein-coding genes were identified in Hap1, and 39,707 in Hap2, with a gene density of 80.62 genes per Mb for Hap1, and 80.16 genes per Mb for Hap2 respectively. The average lengths of these genes were determined to be 3.90 kb and 3.96 kb for the two haplotypes, respectively. Furthermore, there were identified to be average of 5.26 and 5.28 exons per gene (Table 3, Fig. S1).

**Table 3:** Statistics of protein-coding genes in pear cultivar ‘Danxiahong’ genome.

Type	Hap1	Hap2
Gene density (gene/Mb)	80.62	80.16
Gene number	39,936	39,707
Average gene length (bp)	3,904.57	3,958.88
Average CDS length (bp)	1,234.77	1,230.61
Average exon per gene	5.26	5.28
Average exon length (bp)	287.61	286.42
Average intron length (bp)	561.29	571.6

### Repetitive sequence annotation

Repetitive sequences, including tandem repeats and interspersed repeats (transposable elements, TEs) were identified in both assembled haplotype genomes. The tandem repeats were annotated using Tandem Repeats Finder (TRF, v4.09.1)^26^, while the identification of interspersed repeats (transposable elements, TEs) involved a combination of *de novo* and homology-based methods. The LTR-RTs were detected at the DNA level using LTR_FINDER (v1.0.7)^38^. A comprehensive *de novo* repeat library was generated with RepeatModeler (v2.0.1)^39^, after which RepeatMasker (v4.1.2)^40^ was employed to search against both the Repbase TE library^41^ and the *de novo* repeat library. Additionally, RepeatProteinMask (v1.36) was utilized to search against the TE protein database at the protein level.

A total of 305.96 Mb and 311.09 Mb repetitive sequences were identified for Hap1 and Hap2 assemblies, respectively, accounting for approximately 61.77% and 62.02% of the two haplotypes. The predominant types among these repetitive sequences are long terminal repeats (LTR) and DNA elements (Table S1).

### Functional annotations

For functional annotations, comprehensive databases were utilized for the alignments, including the National Center for Biotechnology Information (NCBI), Non-Redundant (NR), Kyoto Encyclopedia of Genes and Genomes (KEGG) database, Gene Ontology (GO), TrEMBL and Swiss-Prot protein databases. Diamond BLASTP (v2.0.7) was employed for the alignments with a threshold of 1E-5. The InterProScan (v5.50-84.0)^42^ was applied to annotate protein domains based on the InterPro protein databases. A total of 98.03% (39,149 out of 39,936) and 97.99% (38,909 out of 39,707) of protein-coding genes were successfully annotated in the aforementioned databases for Hap1 and Hap2, respectively (Table 4).

**Table 4:** Functional annotation information of genes from the haplotypes.

Database	Number-Hap1	Percent (%)-Hap1	Number-Hap2	Percent (%)-Hap2
InterPro	28,461	71.27	28,396	71.51
GO	30,869	77.3	30,750	77.44
KEGG_ALL	37,171	93.08	37,055	93.32
KEGG_KO	13,821	34.61	13,837	34.85
Swissprot	27,135	67.95	27,109	68.27
TrEMBL	39,068	97.83	38,820	97.77
NR	39,119	97.95	38,885	97.93
Annotated	39,149	98.03	38,909	97.99
Unannotated	787	1.97	798	2.01
Total	39,936		39,707	

For the prediction of different types of non-coding RNA, various software tools were utilized. Based on the structural features of tRNA, the software tRNAscan-SE (v2.0.9)^43^ was applied to predict tRNAs in the genome using default parameters. The software RNAmmer (v1.2)^44^ was employed to identify rRNAs in the genome. For miRNA and snRNA prediction, the covariance analysis model from the Rfam database^45^ along with infernal (v1.1.4)^46^ were applied to predict miRNAs and snRNAs in the genome. In total, 3267 non-coding RNAs (152 miRNA, 709 tRNA, 989 rRNA and 428 snRNA) and 3243 non-coding RNAs (155 miRNA, 707 tRNA, 985 rRNA and 411 snRNA) were identified in Hap1 and Hap2 genomes, respectively (Table S2).

## Data Records

The raw genomic sequencing data, including PacBio HiFi, Hi-C, and ONT, has been deposited in the Genome Sequence Archive^47^ at the National Genomics Data Center (NGDC)^48^ under the BioProject number of PRJCA031272. The accession numbers of ONT sequencing data, PacBio HiFi sequencing data, and Hi-C sequencing data are publicly accessible as CRA019931^49^, CRA019932^50^, and CRA019933^51^ respectively. The full-length RNA-seq data has been deposited in the GSA database with the accession number of CRA019930^52^. Furthermore, the raw sequencing data are also deposited in the NCBI under the BioProject number of PRJNA1211178. The accession numbers of ONT sequencing data, PacBio HiFi sequencing data, Hi-C sequencing data, and RNA-seq data are accessible under the Sequence Read Archive number of SRR32016023-SRR32016026^53–56^. The results of genome assembly and the gene annotation files have been deposited in the figshare database^57^. The final chromosome assemblies are available in the NCBI GenBank database under BioProject ID of PRJNA1254322 and PRJNA1254321, with accession number of JBNHTQ000000000 for Hap1 and JBNHTR000000000 for Hap2^58,59^.

## Technical Validation

Complementary approaches were applied to assess the quality of the genome assembly. Firstly, genome completeness was evaluated based on conserved plant genes in the embryophyta_odb10 database of Benchmarking Universal Single-Copy Orthologous (BUSCO) (v5.2.2)^60^. The evaluation of genome completeness revealed that Hap1 and Hap2 had BUSCO scores accounting for 98.8% and 99.0%, respectively, encompassing both single-copy and duplicated BUSCOs (Table 5). The clean BGI paired-end short reads and TGS long-reads (ONT and PacBio HiFi reads) were aligned to each haplotype of the assembled genome using BWA (v0.7.17)^61^ and minimap2 (v2.24)^62^ respectively. Subsequently, the mapping rates, sequencing depth and coverage were evaluated using SAMtools (v1.14). The analysis of mapping rates and sequencing depth revealed that 99.34%-99.97% of the reads were successfully mapped to the two haplotypes, with average sequencing depths ranging from 61.46 to 61.83 for long reads, and from 111.32 to 123.66 for short reads, respectively. Coverage analysis at thresholds of 5×, 10× and 20× demonstrated a high coverage rate ranging from 99.27% to 100% for both haplotypes (Table S3).

**Table 5:** Statistics analysis of BUSCO assessment for protein-coding genes in ‘Danxiahong’ pear.

Statistic	Hap1-Assembly	Hap2-Assembly	Hap1-Annotation	Hap2-Annotation
Complete BUSCOs (%)	1595 (98.82%)	1591 (98.57%)	1592 (98.64%)	1590 (98.51%)
Complete and single-copy BUSCOs (%)	1052 (65.18%)	1051 (65.12%)	1021 (63.26%)	1025 (63.51%)
Complete and duplicated BUSCOs (%)	543 (33.64%)	540 (33.46%)	571 (35.38%)	565 (35.01%)
Fragmented BUSCOs (%)	0 (0)	3 (0.19%)	8(0.50%)	8 (0.50%)
Missing BUSCOs (%)	19 (1.17%)	20 (1.24%)	14 (0.88%)	16 (0.99%)
Total BUSCO groups searched (%)	1614 (100%)		1614 (100%)	

LTR_FINDER^38^ and ltrharvest^63^ were employed to predict the presence of LTRs in the genome, and LTR_retriever (v1.0.7)^64^ was applied for the identification of LTRs and calculation of the LTR assembly index (LAI) for assessing genome assembly quality. The LAI values obtained were 21.94 for Hap1 and 21.28 for Hap2 (Table 2).

The consensus quality (QV) value and completeness of the genome assembly were assessed using Merqury (v1.3)^65^. The results revealed that Hap1 and Hap2 had a quality value (QV) of 40.6567 and 40.6569, respectively (Table 2). Overall, these findings indicate the high quality of the ‘Danxiahong’ pear cultivar genome assembly.

All functional annotations have been comprehensively supplemented in Tables S4, S5, providing detailed annotations for every gene within the haplotype-resolved genome. The syntelogous genes between Hap1 and Hap2 in the Danxiahong genome assembly was analyzed. The detailed list of syntelogous gene pairs is provided in Table S6. Additionally, unique genes for each haplotype are annotated in Supplementary Tables S7, S8, with functional descriptions, chromosome positions and enriched pathways. These annotations and analyses validate the high quality of haplotype-resolved genome assembly and provide a valuable resource for investigating haplotype-specific regulatory mechanisms and allele-specific expression.

## Supplementary information

Supplementary Fig S1-Table S1-S3

Supplementary Table S4-S8

## Data Availability

No specific code or script was developed in this work, and all the bioinformatics software utilized and their corresponding version were described in detail within the Methods section. For the specific parameters that were not mentioned for the bioinformatics software, the analysis was performed using default parameters as suggested by developers.
