# Supplementary material for: Haplotype-resolved T2T genome assembly of the pear cultivar ‘Danxiahong’
Source: Sci Data. 2025 Jun 18;12:1024. doi: 10.1038/s41597-025-05380-3 (PMC12177082; doi:10.1038/s41597-025-05380-3)
Supplement: Supplementary file 1 — Supplementary Fig S1-Table S1-S3 [file 41597_2025_5380_MOESM1_ESM.docx]

**Table S1.** Statistics analysis of the repetitive elements in two haplotypes of 'Danxiahong' pear.

| **Type** | **Hap1** | | **Hap2** | |
| --- | --- | --- | --- | --- |
|  | **Combined TEs Length (bp)** | **Combined TEs % in genome** | **Combined TEs Length (bp)** | **Combined TEs % in genome** |
| DNA | 76,983,478 | 15.54 | 76,736,372 | 15.3 |
| LINE | 10,964,163 | 2.21 | 11,395,373 | 2.27 |
| SINE | 198,487 | 0.04 | 152,646 | 0.03 |
| LTR | 215,434,762 | 43.49 | 220,544,913 | 43.97 |
| Other | 511 | 0 | 663 | 0 |
| Unknown | 15,498,590 | 3.13 | 14,884,835 | 2.97 |
| Total TE | 305,968,967 | 61.77 | 311,090,894 | 62.02 |

**Table S2**. Summary of the annotated non-coding RNAs in different haplotypes of pear cultivar 'Danxiahong'.

| **ncRNA** | **Type** | **Number-Hap1** | **Average length (bp)-Hap1** | **Total length (bp)-Hap1 (% of genome)** | **Number-Hap2** | **Average length (bp)-Hap2** | **Total length (bp)-Hap2 (% of genome)** |
| --- | --- | --- | --- | --- | --- | --- | --- |
| miRNA | — | 152 | 128.05 | 19,464（0.003929） | 155 | 127.88 | 19,822（0.003952） |
| tRNA | — | 709 | 75.38 | 53,446（0.010789） | 707 | 75.37 | 53,287（0.010623） |
| rRNA | rRNA | 989 | 319.57 | 316,057（0.063803） | 985 | 248.76 | 245,027（0.048849） |
|  | 18S | 26 | 1,806.92 | 46,980（0.009484） | 13 | 1,806.92 | 23,490（0.004683） |
|  | 28S | 27 | 5,986.00 | 161,622（0.032627） | 18 | 6,220.89 | 111,976（0.022324） |
|  | 5S | 936 | 114.80 | 107,455（0.021692） | 954 | 114.84 | 109,561（0.021842） |
| snRNA | CD-box | 260 | 105.19 | 27,349（0.005521） | 249 | 105.59 | 26,292（0.005242） |
|  | HACA-box | 31 | 127.87 | 3,964（0.0008） | 29 | 127.21 | 3,689（0.000735） |
|  | splicing | 137 | 139.74 | 19,145（0.003865） | 133 | 139.33 | 18,531（0.003694） |

**Table S3**. Analysis of mapping rates and sequencing coverage across different haplotype genomes.

| Haplotype | Data_type | Mapping_rate (%) | Average_sequencing_depth | Coverage (%) | Coverage (>= 5X, %) | Coverage (>= 10X, %) | Coverage (>= 20X, %) |
| --- | --- | --- | --- | --- | --- | --- | --- |
| Hap1 | BGI | 99.34 | 123.66 | 99.82 | 99.7 | 99.59 | 99.31 |
|  | HIFI | 99.92 | 111.32 | 100 | 100 | 100 | 99.98 |
|  | ONT | 99.97 | 61.83 | 100 | 100 | 99.99 | 99.67 |
| Hap2 | BGI | 99.38 | 122.36 | 99.82 | 99.7 | 99.58 | 99.27 |
|  | HIFI | 99.77 | 112.36 | 100 | 100 | 100 | 99.96 |
|  | ONT | 99.73 | 61.46 | 100 | 100 | 99.99 | 99.57 |


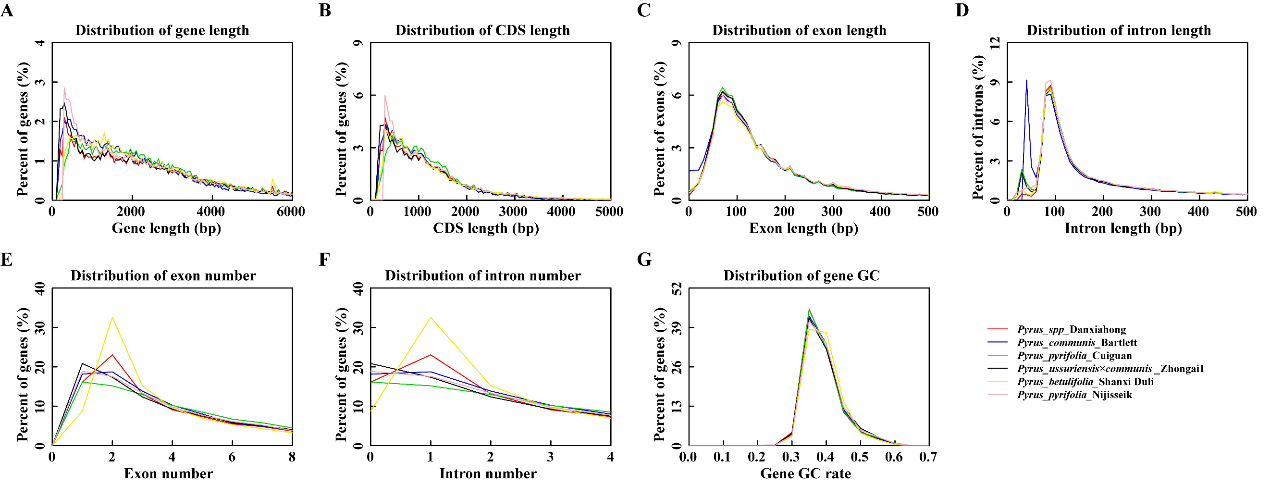


**Fig. S1** The composition of gene elements in the pear cultivar ‘Danxiahong’ genome with other Pyrus species. (A) Distribution of gene length with other *Pyrus* species. (B) Distribution of CDS length with other Pyrus species. (C) Distribution of exon length with other Pyrus species. (D) Distribution of intron length with other Pyrus species.
